# Leader's Humility: Unveiling the Mediating Effect of Decent Work on the Relation Between Humble Leadership and Nurses' Grit

**DOI:** 10.1155/jonm/4640687

**Published:** 2025-06-20

**Authors:** Ahmed Abdellah Othman, Sally Mohammed Farghaly Abdelaliem, Hossam Mohamed Mahran, Hind Ismail Ali

**Affiliations:** ^1^Department of Nursing Administration, Faculty of Nursing, Sohag University, Sohag City, Egypt; ^2^Department of Nursing Management and Education, College of Nursing, Princess Nourah bint Abdulrahman University, P.O. Box 84428, Riyadh 11671, Saudi Arabia; ^3^Department of Nursing, College of Applied Medical Sciences in Wadi Addawasir, Prince Sattam bin Abdul Aziz University, Wadi Addawasir, Saudi Arabia

**Keywords:** decent work, humble leadership, leader's humility and nurses' grit

## Abstract

**Background:** Humble leadership emerges in various contexts and circumstances throughout time; it is dynamic and essential in motivating staff nurses to achieve their objectives by influencing and mentoring them with humility, thereby creating an environment that encourages their proactive and creative work practices.

**Aim:** This study aim to assess the relation between humble leadership and nurses' grit and also examine the mediating effect of decent work on the relation between humble leadership and nurses' grit.

**Methods:** This was a cross-sectional study of nurses in Sohag University Hospital, Egypt. A total of 454 nurses who had worked at their employing facility for at least 6 months participated in this study. Study variables included humble leadership, nurses' grit, and decent work.

**Results:** Decent work mediated the relationship between humble leadership (*B* = 0.267, *t* = 4.968, and *p* < 0.001) and nurses' grit. Also, there was a statistically significant positive correlation between the participant nurses' perception of humble leadership and decent work (0.386, *p* < 0.001), nurses' perception regarding grit with decent work (*r* = 0.421 and *p* = 0.000), and humble leadership (*r* = 0.363 and *p* < 0.001).

**Conclusions:** The importance of addressing decent work to enhance nurses' grit and promote perception of humble leadership. This study suggests that not only humility by leadership is sufficient to enhancing the nurses' grit as consistent interest and persistent effort but also the decent work have vital role in pushing the nurses for more commitment and interest in the work of goal achievement.

## 1. Introduction

In healthcare settings, leadership plays a crucial role in shaping the work environment, influencing not only the overall quality of care but also the wellbeing and performance of healthcare professionals [1]. One leadership style that has garnered increasing attention is humble leadership, characterized by humility, openness to feedback, and a focus on empowering others. This approach is seen as particularly impactful in nurturing an organizational culture of trust, collaboration, and support—essential components for the demanding and high-pressure work that nurses encounter daily [2].

Grit, which is characterized as tenacity and enthusiasm for long-term objectives, is a significant predictor of personal resilience and consistent effort in demanding work settings [3]. Particularly, nurses frequently deal with severe pressures, such as physical and emotional tiredness and the difficulty of upholding patient care standards within strict limitations. Therefore, healthcare organizations must comprehend how grit may be developed and maintained [4, 5].

In this context, the concept of decent work—encompassing fair wages, safe working conditions, respect, and opportunities for personal growth—emerges as a potential mediator in the relationship between leadership and nurses' grit. Decent work not only contributes to a positive work environment but also influences employees' motivation, engagement, and long-term commitment [6].

## 2. Background

Humility is the capacity to view oneself honestly and impartially. This implies that in the workplace, one needs to be able to acknowledge one's advantages and successes in addition to being aware of one's weaknesses and limits. Some of the many positive outcomes for employees that humble leadership is associated with is work engagement [7–9], organizational citizenship behavior (OCB) [10, 11], and voice behavior [12].

A person's level of humility is a reflection of his or her intentions to act in a certain humble manner and his or her desire to be humble in his or her relationships with others [13]. Humility conforms with the Theory of Reasoned Actions integration of cognitive beliefs, emotional attitudes, conative intentions, and actual behaviors [14]. Also, there were different theories that explained leadership humility: the servant leadership theory (SLT) by Greenleaf [15], the absorptive capacity theory (ACT) by Cohen and Levinthal [16], and self-determination theory (SDT) by Deci Ryan [17].

The behavior-based authority style of humble leadership is composed of three key measurements [18, 19]: (a) shown a willingness to accurately view oneself, paying close attention to one's special qualities and limits. (b) A passion for the qualities and commitment of others; respect the qualities and commitment of others instead of feeling inferior to them. (c) Showing openness to direction and the capacity to take in criticism, fresh insights, and information from others [20].

Humble leadership is characterized by self-awareness, openness, and appreciation for others. This leadership style is underexplored in healthcare. Nurses who work under humble leaders feel more likely to be motivated to go beyond their prescribed duties, take initiative, and seek creative solutions to problems [13]. A humble leadership style impacts healthcare settings where innovation and creativity can thrive and can significantly impact psychological safety. When nurses perceive their leaders as humble, they are likelier to cooperate, speak up, and share ideas with their colleagues. Psychological safety enhances patient outcomes by encouraging open communication, teamwork, and nurse collaboration [2, 21].

Nurses have faced many challenges, including job insecurity, concerns about their families' health, increased workloads, unpredictable shifts, increased risk of infection, inadequate protective equipment, social stigma, and stagnant pay [22]. One of the most crucial things health organizations can do to prevent this decline in work conditions and its impact on nurses is to promote decent work conditions [23]. The concept of decent work includes workplace security, fair compensation, social protection for families, opportunities for social integration and personal development, the freedom to express concerns and take part in life-altering decisions, and equal treatment and opportunity for all people, regardless of gender [24, 25].

Working theory psychology (PWT) maintains that decent work is influenced by external variables such as financial constraints and marginalization. Furthermore, there are psychological traits, such as work acceptance and decision-making autonomy, that serve as mediating variables [26]. Previous research has identified important elements of decent work. Five characteristics of decent work have been proposed by Mao and others [27]: work incentives, work position, work culture, work progress, and work recognition. Within an organization, having access to quality work can lead to a number of beneficial outcomes, including engagement, job satisfaction, and a sense of purpose. It can also inspire passion for achieving goals and the “grit” that comes from overcoming obstacles [28, 29].

Duckworth and colleges [30] introduced the idea of grit as trait-level determination and enthusiasm for long-term goals and stated that grit refers to the determination of an individual to keep working hard even in the face of setbacks and represent the tendency of an individual to have a positive interest in constant goals. Grit plays an essential role in nurses' academic performance, mental health, work ability, job satisfaction, and overall performance, which may also reduce staff turnover rate. The theory on the relationship between grit and health [31] holds that grit is a protective factor for people dealing with stressful events. Grit is a crucial quality that affects a variety of nursing performance areas and makes them more gregarious, which may enhance performance. Nurses with low grit have been found to have higher stress levels and more mental health problems, such as anxiety, depression, and other harmful emotions [32, 33].

According to Park and Cho [34], those with a high degree of grit continuously show interest in their goals, put in a lot of effort, and adjust their behavior as necessary to reach their objectives. Grit has five essential qualities that are also necessary for successful leadership. These are a few of them: (1) courage: the ability to take aggressive, positive action in the face of adversity. (2) Conscientiousness or the will to succeed even in the face of little victories. (3) Long-term goals: the ability to focus on and sustain the tenacity required to achieve longer-term goals. (4) Resilience: the ability to use adversity as a springboard for improvement. (5) Excellence is the ability to strive for greatness without anticipating success [35].

The concept of humble leadership and the workplace has been the subject of numerous recent scholarly studies [2]. While there has been some discussion about the value of decent work for nurses, it is crucial to remember that there are not any comprehensive studies in the field of nursing research that examine the concept of decent work for registered nurses and how it affects their fortitude and humility as leaders. Therefore, this study aims to assess the association between nurses' grit and perception of humble leadership, as well as to look into the role that decent work serves as a mediator in the relationship between nurses' grit and perception of humble leadership.

### 2.1. Study Objectives

1. Measure mean score of the nurses' perception of humble leadership, decent work, and grit among nurses in the work setting
2. Assess the association between nurses' grit and perception of humble leadership among nurses
3. Evaluate the role of decent work serves as a mediator in the relationship between nurses' grit and perception of humble leadership.

## 3. Methods

### 3.1. Design, Setting, and Participants

This research utilized a descriptive cross-sectional design, adhering to the guidelines established by the Strengthening the Reporting of Observational Studies in Epidemiology (STROBE). The study was conducted at the Sohag University Hospital located in Sohag Governorate, Egypt.

The study focused on nurses affiliated with the university hospital at Sohag University. To determine the appropriate sample size, researchers utilized G∗Power software, concluding that 454 participants were necessary to achieve a statistical power of 0.90 for *t*-test analyses. Using a point biserial correlation model, the significance level (*α*) was set at 0.05. The study assumed a moderate effect size of 0.15, in line with findings reported by Xue et al. [24]. Eligibility criteria included (1) holding a valid nursing license and currently working in a clinical setting; (2) having at least 6 months of continuous experience within the same department; and (3) willingness to participate, indicated through informed consent.

To minimize selection bias, the researchers anonymized the entire nursing population by converting their names into numerical identifiers. Using the SPSS software's “Select Cases” function and the “Random Sample of Cases” method, participants were randomly chosen. The required sample size was predetermined by the researcher, while the software handled the random selection process autonomously, eliminating the potential for manual influence or bias.

### 3.2. Study Instruments

#### 3.2.1. Demographic Form

It included data such as age, gender, educational level, place of residence, marital status, years of experience, and perception of monthly income.

#### 3.2.2. Leader Humility Scale

It was employed by Owens et al. [36] to gauge nurses' opinions of their nurse leader's humility. It consisted of nine items divided into three dimensions as follows: willingness to see oneself accurately (3 items), appreciation of others' strengths (3 items), and contributions and teach ability (3 items). A 5-point Likert scale was used to rate the scale's components (1 being strongly disagreed and 5 being strongly agreed), with a higher score denoting a higher degree of nurses' opinion about their nurse leader's humility. The scale exhibited internal consistency reliability of 0.94 [36] and *α* = 0.74 [37]. The study demonstrated high internal consistency, evidenced by a Cronbach's alpha score of 0.877, which signifies robust reliability. After translating the leader humility scale from English to Arabic, the researchers carried out an exploratory factor analysis (EFA). Before rotation, factor loadings fell between 0.416 and 0.635, which improved to a range of 0.563–0.877 following varimax rotation. All loading values surpassed the minimum acceptable threshold of 0.36, cumulatively explaining 67.847% of the total variance. Furthermore, the Kaiser–Meyer–Olkin (KMO) measure indicated excellent sampling adequacy with a value of 0.875, confirming the dataset's appropriateness for factor analysis.

#### 3.2.3. The Decent Work Perceptions Scale (DWPS)

It was developed by Chinese scholars and has been widely used among nurses [27]. It was utilized in this study to measure the level of perceived decent work among nurses. It included five dimensions, which add up to 16 items: work rewards (4 items), work position (3 items), career development (3 items), work recognition (3 items), and work atmosphere (3 items). The responses were scored between 1 and 5 on a 5-point Likert scale that went from “completely disagree” to “completely agree.” Higher scores indicated a stronger impression of good work among nurses. The total score varied from 16 to 80. The scale's Cronbach's *α* coefficient was determined to be 0.960. The dimensions' Cronbach's *α* values were as follows: career development 0.859, work rewards 0.916, work position 0.837, work recognition 0.946, and work atmosphere 0.899 [27] and 0.939 [38].

The study demonstrated high internal consistency, evidenced by a Cronbach's alpha of 0.875, suggesting strong scale reliability. Following the English-to-Arabic translation of the DWPS, researchers performed an EFA to assess content validity. Initial factor loadings ranged from 0.460 to 0.960 and, after applying varimax rotation, improved to between 0.513 and 0.870—all surpassing the acceptable threshold of 0.37. These components accounted for 75.50% of the total variance. The KMO test yielded a value of 0.920, indicating outstanding sampling adequacy, while Bartlett's test of sphericity was highly significant (*p* < 0.001), affirming that the correlation matrix was suitable for factor analysis. As a result, every item in the scale was retained.

#### 3.2.4. Grit Scale

It was developed by Duckworth and Quinn [39] to measure grit. It is an 8-item, 5-point Likert scale was used; higher scores indicate greater grittiness. The two unique components of grit, according to this scale, are consistent interest and persistent effort. According to Duckworth and Quinn [39], the reliability of the scale was 0.77.

The study established strong internal consistency for the Arabic version of the Grit Scale, with a Cronbach's alpha of 0.894, indicating high reliability. To assess content validity after translation from English, researchers employed EFA. Prior to rotation, factor loadings ranged from 0.480 to 0.960 and improved to 0.520 to 0.891 following varimax rotation—well above the acceptable threshold of 0.42. These components together accounted for 76.32% of the total variance. The KMO statistic was 0.930, reflecting excellent sample adequacy for factor analysis. Furthermore, Bartlett's test of sphericity was highly significant (*p* < 0.001), supporting the suitability of the correlation matrix for this analysis. As a result, all scale items were retained.

### 3.3. Instrument Translation and Validation Process

The work field included the main two phases; tool translation to Arabic to be simple for all employees in primary healthcare center and the second phase include tool validation.

#### 3.3.1. Phase 1

The instrument initially involved the standard translation process. The forward–backward translation, which is the most commonly applied translation process for questionnaires or inventories, was performed [40]. In the first step of the procedure, the original English of the Leader Humility Scale, DWPS, and grit nurses scale were translated into Arabic language by two experienced translators and assessment of forward translation drafts was performed by two other researchers to review each translated item independently and choose the most adequate in terms of clarity, common language, and cultural diversity. The second step included retranslation of the agreed Arabic text to English language by a researcher who had not previously seen the original version. The backward translation was compared with the original version of the survey, and judgments about the inaccuracies were made by two other researchers.

#### 3.3.2. Phase 2

The instrument of choice was validated through several steps, including expert reviews, a pretest among a small sample of respondents, a quantitative study, and a test–retest survey. The content validity was done through expert reviews. The instrument was sent to experts for review, including sociologists, administrative staff working at the hospital, public health specialist, and officer from Ministry of Health. It was then revised based on their comments. The content validity of the instrument will be assessed using the Content Validity Index (CVI) as suggested by Polit et al. [41].

The face validity was done through pretest. A group discussion with administrative staff working at the hospital was conducted in order to ensure that items will be phrased in a culturally acceptable manner [42]. They were asked to give comments on the instrument in terms of wording and meaning of the items. The construct validity was done through a quantitative study among number administrative staff at the hospital for assessment of the instrument's reliability and validity. EFA is one useful method to identify the number of constructs that might exist among a group of items [43]. Internal consistency reliability to estimate the reliability of a scale is calculating the internal consistency coefficient, as an indicator of how well the single items of an instrument reflect a common, underlying factor [44]. In this study, the internal consistency reliability of each construct of the questionnaire will be done by calculating the coefficient alpha [45]. Test–retest reliability method was used to establish the temporal stability of a scale through Pearson correlation. It indicates scale consistency over time. The test can be done by administering the same scale on a group of respondents at two different points of a time interval.

### 3.4. Data Collection Procedures

Data collection for this study was conducted from August to October 2024. A pilot research was carried out with 45 nurses to evaluate the instruments' reliability, relevance, and intelligibility. Because the results of the pilot study showed that no modifications were required and confirmed that the instruments were appropriate for the primary research, these participants were added to the main study. The process commenced after obtaining the necessary permissions and securing Excel spreadsheets containing the details of all nurses from the hospital's human resources department. Participants were selected using a random number generator in an Excel spreadsheet by determining the sampling frame (potential respondents) and repeating the process until the required number of nurses from each unit was achieved. Before data collection, researchers provided each nurse with a detailed explanation of the study objectives, emphasizing that participation was entirely voluntary. Written informed consent was obtained from all participants as a prerequisite for their involvement. To ensure confidentiality and foster trust, researchers assured participants that their responses would remain confidential. Questionnaires were distributed in different shifts: morning, evening, and night to the workload of the units from Saturday to Thursday. On average, participants spent 15–20 min completing each questionnaire.

#### 3.4.1. Ethical Considerations

The Research Ethics Committee of the Faculty of Nursing at Sohag University in Egypt provided ethical permission for the study (serial no: 180). The rights and safety of the participants were protected because the study complied with all applicable laws, local regulations, and the Declaration of Helsinki's ethical guidelines. The goals of the study were fully explained to the participants, who also received assurances that their participation would be completely voluntary and confidential. They received assurances that all information gathered would be kept confidential and that only members of the approved study team would have access to it. Before any data were collected, written informed consent was obtained from each participant.

### 3.5. Data Analysis

Data analysis was performed using SPSS 26.0 (IBM Inc., Chicago, IL, USA) to evaluate the survey responses from the 454 nurses. The participants' general characteristics and the scores obtained on various scales. Descriptive statistics were evaluated for frequencies (No./%) and mean ± standard deviations (S.D.) and were utilized to summarize. *T*-tests and ANOVA tests are used to measure the relation between variables and participants' demographic data and are typically used to compare mean differences among groups. The correlations between humble leadership, decent work, and nurses' grit were examined by Pearson's correlation analysis utilized to assess. JASP 0.14.1.0 was used to test the mediating role of decent work between humble leadership and nurses' grit.

## 4. Results

### 4.1. Relationship Between Total Study Variables and Participant Nurses' Characteristics

Table 1 shows that 70.7% of the participant nurses had age ranging from 25 to 35 years old, 80.4% were female, 72% had diploma/nursing technical institute, 61.7% were from rural area, 80.2% were married, 60.4% had 5 ≤ 10 years of experience, and 64.2% perceived that they had enough income monthly. Also, the findings clarify that there was a statistically significant difference between participant nurses' perceptions of decent work and their age at (*p* < 0.001) with a high mean (3.16 ± 0.89) at age 35 ≥ 40 years old, gender at (*p* = 0.041) with a high mean (3.07 ± 0.891) related to female, years of experience at (*p* < 0.001) with a high mean (3.19 ± 0.88) related to 10 ≥ years of experience, and perception of monthly income at (*p* = 0.004) with a high mean (3.77 ± 0.62) related to enough income. Regarding grit, there was a statistically significant difference between participant nurses' grit and their educational level at (*p* = 0.001) with a high mean (3.24 ± 0.94) related to postgraduate education, participants' years of experience at (*p* = 0.008) with a high mean (3.23 ± 0.854) related to 10 ≥ years of experience, and participants' perception of monthly income at (*p* = 0.002) with a high mean (3.87 ± 0.73) related to enough monthly income.

### 4.2. Descriptive Analysis

Table 2 reveals that the mean score of the participant nurses regarding humble leadership was (2.84 ± 0.68), willingness to see oneself accurately (2.92 ± 1.16), appreciation of others' strengths (2.93 ± 1.18) and contributions, and teach ability (2.60 ± 0.98). Regarding the mean score of decent work, it was (2.94 ± 0.86) and its subscale, work rewards (2.15 ± 0.97), work position (2.34 ± 0.98), work atmosphere (2.48 ± 1.11), work development (2.40 ± 1.07), and work recognition (2.48 ± 1.22). In addition, the mean score for the participant nurses' grit was (2.78 ± 0.89).

### 4.3. Correlations of Variables

Table 3 clarifies that there was a statistically significant positive correlation between the participant nurses' perception of humble leadership and decent work (0.38, *p* < 0.001). Besides that, there was a statistically significant correlation of the participant nurses' perception regarding grit with decent work (*r* = 0.42 and *p* < 0.001) and humble leadership (*r* = 0.36 and *p* < 0.001).

### 4.4. Mediation Analysis

**Figure 1:**
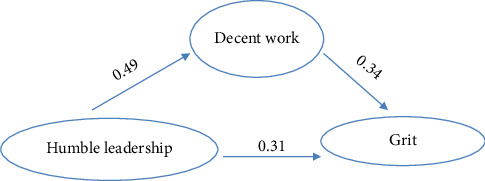
Path mediation analysis of participant nurses' decent work between humble leadership and grit (*N* = 454).

Table 4 and Figure 1 illustrate that there was a statistically significant direct effect of humble leadership on decent work at (*B* = 0.488, *t* = 8.89, and *p* < 0.001), on nurses' grit at (*B* = 0.308, *t* = 5.26, and *p* < 0.001), and of decent work on nurses' grit at (*B* = 0.342, *t* = 7.35, and *p* < 0.001). Furthermore, there was a statistically significant indirect effect of humble leadership on nurses' grit when the decent work act was a mediator variable at (*B* = 0.267, *t* = 4.96, and *p* < 0.001).

## 5. Discussion

Creating work environments that support optimal health and wellbeing for nurses is essential to achieving optimal organizational performance. Sustaining the physical and mental wellbeing of nurses requires a positive work environment [46]. Humble leaders who appreciate staff nurses' expertise, establish rapport with them, and recognize their contributions and talents foster a pleasant team environment and enhanced collaboration—a place where direction and support are vital [47]. Therefore, the current study aimed to assess the relation between humble leadership and nurses' grit and examine the mediating effect of decent work between humble leadership and nurses' grit.

Furthermore, the current study findings clarified that there was a statistically significant difference between participants' perceptions of decent work and their age, gender, years of experience, and monthly income. Regarding grit, there was a statistically significant difference between nurses' grit and their educational level, years of experience, and monthly income. Accordingly, Mohamed et al. [48], El‐Gazar et al. [49], and Carbone [50] in those studies revealed that there were statistically significant relationships present between grit and years spent in the field as well as age. In addition, these results agreed with Xue et al. [24], who found that age and years of working emerged as significant influencing factors affecting decent work. In addition, Li et al. [9], in their work, suggested that years of work experience effect the decent work among psychiatric nurses. This may be explained by improved nurses' cognitive abilities and adherence to ethical values and commitment to their work and goals with older age and increased years of experience. In addition, with an appropriate amount of income, their enthusiasm and commitment to their goals increase to achieve a better life.

With regard to descriptive analysis, the mean scores of the participants' nurses regarding humble leadership were 2.8488 ± 0.684, willingness to see oneself accurately 2.9251 ± 1.161, appreciation of others' strengths 2.931 ± 1.183, and contributions and teach ability 2.6035 ± 0.9817. These results were supported by the study conducted by Spector [43], which revealed that the majority of nursing managers have a moderate level of humble leadership from staff nurses' perspective. In addition, these results were in the same line as El‐Gazar et al. [49], who found that the mean scores of the participants' nurses regarding humble leadership were 3.08 ± 1.01, willingness to see oneself accurately 2.92 ± 1.10, appreciation of others' strengths 3.17 ± 1.04, and contributions and teach ability 3.13 ± 1.04. This may be due to leaders' perception that practicing humility will make followers perceive them as weak leaders with low self-esteem, in addition to continuous change in the healthcare environment that may affect the workplace negatively.

The current study showed that the mean score for the participants' nurses' grit was 2.786 ± 0.8949. This finding reflects that participant nurses had a near-neutral perception of grit in their work environment and were hardly able to feel empowered and confident in their abilities. Also, El-Gazar et al. reported that the mean overall score for participants' experience of grit at work was neutral. Moreover, these results disagreed with Burke et al. [45], who demonstrated that graduate nursing students had high levels of grit as measured by the Grit-S (*M* = 3.9 and SD = 0.5). This revealed that grit to work developed during years of contact at work [49].

Regarding the mean score of decent work, the participant nurses' perception was hardly near to the midpoint, which indicated a low level of decent work conditions. Regarding decent work subscales, work rewards, work position, work atmosphere, work development, and work recognition were near neutral levels. This result was in the same line with a previously mentioned study by El-Gazar et al. [49], where the mean overall score for participants' experience of decent work was neutral (48.82 ± 12.09). The study of Wang et al. [51] found that the nursing students perceived decent work groups scored at a low level in all dimensions. On the opposite side, these results were different with the study performed by Ragadu and Rothmann [52], which demonstrated that the mean score of decent work and adequate compensation was 4.85 ± 1.60. Also, Zhang et al. [53] found that the mean score of decent work was 55.68 ± 10.84.

According to Mrayyan [2], humble leadership in nursing fosters a positive work atmosphere, enhances patient care quality, and improves team relations. It encourages mutual respect, collaboration, and continual development, which are essential for the success of the nursing profession. In addition, humble leadership cultivates and strengthens nurses' grit by creating conducive work environments that promote nurses' engagement, creativity, and innovation; providing support; exhibiting emotional intelligence; fostering a sense of purpose; and encouraging a growth mindset [54].

Regarding correlation between study variables, the current study clarified that there was a statistically significant positive correlation between humble leadership and decent work and between humble leadership and nurses' grit. Also, there was a statistically significant correlation between the participants' experience of decent work and grit. These results were similar with El-Gazar and her colleagues [49], who revealed that the experience of decent work had a positive correlation with nurses' grit. In addition, according to Luo et al. [54], their meta-analysis about “Humble Leadership and Its Outcomes” revealed that humble leadership is positively related to affective commitment, which helps better achieve the missions and goals of hospitals, affective trust, creativity, engagement, job satisfaction, organizational identification, self-efficacy, task performance, and voice. Moreover, Michalec et al. [55], in their study, found that humility and being humble within the clinical setting were seen as fostering psychological safety and building trust among care delivery team members and lending to a positive work environment. This may be attributed to humble leaders' openness to learning, modeling tasks for followers, listening and giving credit to different nurses' viewpoints, offering nurses space to arrange their ways and contents of work, and creating a positive organizational atmosphere that will increase nurses' grit.

Our study illustrated that there was a statistically significant direct effect of humble leadership on decent work, humble leadership on nurses' grit, and decent work on nurses' grit. Furthermore, there was a statistically significant indirect effect of humble leadership on nurses' grit when decent work acts as mediator variables. These results were supported by Chughtai et al. [56], who revealed that humble leaders motivate and inspire their subordinates by giving them awareness and caring about their future growth and development in the form of career success. Moreover, Chandler et al. [57] suggested that subordinates perceived humble leaders as charismatic and influential personalities that motivated them to demonstrate emotional, ethical, and extra-role behaviors. Furthermore, Mrayyan and Algunmeeyn [58] reported that humble leaders create humble team members who create a ‘collective' and positive team climate that focuses on team performance, goals, and higher collaboration.

In addition, these results were compatible with Soyalın [59], who revealed that workplace happiness has a partial mediating role in humble leadership's effect on employee performance. Also, humble leadership not only contributes to happiness and positive emotions in the organization but also increases the performance levels of employees. This may be explained by humble leaders' role in creating a decent workplace that is characterized by security, fair income, opportunities for personal development and social integration, and freedom for individuals to express their concerns, which in turn affects nurses' grit and commitment to their goals and career success.

## 6. Conclusion

The study findings concluded that there was a statistically significant direct effect of humble leadership on decent work and nurses' grit. This revealed that nurses who experience higher humility from leader and head nurses in the presence of decent work tend to be more grit in work workplace. Also, not only the type of leader who motivates and directs the nurses for more energy and work commitment to achieve organizational goals but also the work environment plays a vital role. In summary, fostering humble leadership and decent work environments plays a pivotal role in enhancing nurses' grit, leading to improved patient care outcomes and a more resilient healthcare workforce.

Understanding this mediating role offers insights into enhancing nurses' resilience and commitment, which are crucial for delivering quality patient care. The findings emphasize significant implications for both nursing practice and healthcare management. Enhancing leadership practices through working on providing fairer work rewards, work position career development, work recognition, and work atmosphere. Improving work conditions by improving the work environment where nurses trust their leader's ability to be more supportive and an advocate. Informing policy and organizational strategies by integrating humble leadership training and promoting decent work environments aimed at improving nurse retention and performance. Further studies are needed to explore the causal relationships and potential moderating factors in this dynamic. Longitudinal research could provide deeper insights into how changes in leadership styles and work conditions over time affect nurses' grit and overall wellbeing.

### 6.1. Challenges and Limitations

While this study sheds light on how decent work conditions and a leader's humble approach can foster grit among nurses, it is not without limitations. One key constraint is its cross-sectional design, which restricts the ability to draw conclusions about cause-and-effect relationships. To gain deeper insights into the causal links and evolving dynamics between these factors, future research should adopt longitudinal methodologies. In addition, the study was confined to a single hospital, which limits the generalizability of the findings. Nurses' interpretations of their leader's humility, the organizational culture, and perceptions of decent work may vary significantly depending on individual backgrounds, experiences, and personal coping styles. Moving forward, research should include larger and more diverse samples, employ mixed-methods designs, and account for potential confounding variables to ensure more comprehensive and reliable results.

### 6.2. Implications and Recommendation

In order to foster a work environment that strengthens nurses' grit via humility and decent work, healthcare organizations should put in place focused leadership training programs that emphasize the development of humble leadership competencies. Our findings demonstrate the relationships between nurse grit and humble leadership style in the presence of decent work. Self-awareness, active listening, inclusive decision-making, and receptivity to criticism should all be prioritized in these programs. Organizations can also incorporate humility as a fundamental leadership characteristic into promotion processes and performance reviews.

The creation and implementation of decent work standards, such as equitable workload distribution, pay structures, open recognition processes, and flexible scheduling to support work–life balance, should be part of policy suggestions. Institutions should also set up official avenues for feedback and psychological safety so that nurses can express their concerns and help the organization grow without worrying about reprisals.

Finally, implementing grit-building activities, such as mentoring programs, resilience training, and long-term goal-setting assistance, can strengthen nursing staff members' individual tenacity. Together, these tactics will improve team and individual performance while also fostering a more sustainable and healthy healthcare workplace.

## Figures and Tables

**Table 1:** Relationship between total study variables and studied nurse's characteristics (*N* = 454).

Demographic characteristics	No. (%)/mean	Humility leadership		Decent work		Grit	
		*M* (SD)	t/F (P)	*M* (SD)	t/F (*p*)	*M* (SD)	t/F (*p*)
Age							
< 25	78 (17.2)	2.63 ± 0.41		2.69 ± 0.77		3.07 ± 0.76	
25 < 35	321 (70.7)	2.92 ± 0.743	7.23	2.78 ± 0.65	11.47	3.10 ± 0.88	1.42
35 ≥ 40	55 (12.1)	2.70 ± 0.53	0.34	3.16 ± 0.89	< 0.001^∗∗^	3.31 ± 1.08	0.243
Gender							
Male	89 (19.6)	2.74 ± 0.53	1.65	2.88 ± 0.73	1.84	2.90 ± 0.87	2.69
Female	365 (80.4)	2.87 ± 0.71	0.051	3.07 ± 0.89	0.041^∗^	3.18 ± 0.89	0.437
Education level							
Diploma/Nursing Technical Institute	327 (72.0)	2.84 ± 0.70		3.09 ± 0.861		3.10 ± 0.87	
Bachelor's degree in nursing	113 (24.9)	2.89 ± 0.63	1.25	2.88 ± 0.824	2.72	2.74 ± 0.88	2.40
Postgraduated education	14 (3.1)	2.59 ± 0.58	0.287	2.95 ± 1.164	0.066	3.24 ± 0.94	< 0.001^∗∗^
Place of residence							
Urban	174 (38.3)	2.89 ± 0.69	1.22	3.11 ± 0.87	1.45	3.19 ± 0.90	1.18
Rural	280 (61.7)	2.81 ± 0.67	0.223	2.99 ± 0.85	0.145	3.08 ± 0.89	0.235
Marital status							
Unmarried	90 (19.8)	2.794 ± 0.68	3.47	3.04 ± 0.88	0.05	3.09 ± 0.90	1.743
Married	364 (80.2)	3.07 ± 0.63	0.075	3.03 ± 0.79	0.958	3.27 ± 0.85	0.082
Years of experience							
1 < 5 years	158 (34.8)	2.86 ± 0.64		2.70 ± 0.46		2.96 ± 0.92	
5 ≤ 10 years	274 (60.4)	2.84 ± 0.71	0.50	2.82 ± 0.81	11.30	2.97 ± 0.99	4.90
< 10 years	22 (4.8)	2.71 ± 0.58	0.602	3.19 ± 0.88	< 0.001^∗∗^	3.23 ± 0.854	0.008^∗^
Perception monthly income							
Enough	291 (64.2)	2.86 ± 0.70	0.737	3.77 ± 0.62	0.38	3.87 ± 0.73	1.74
Not enough	163 (35.8)	2.89 ± 0.53	0.461	2.26 ± 0.59	0.004^∗^	3.00 ± 0.68	0.002^∗^

^∗^Statistically significant difference *p* ≤ 0.05.

^∗∗^Highly statistically significant difference *p* ≤ 0.001.

**Table 2:** Descriptive analysis of the study variables among participant nurses (*N* = 454).

Variables	*N*. items	Minimum	Maximum	Mean	SD
Humble leadership	9	1.67	5.00	2.84	0.68
Willingness to see oneself accurately	3	1.00	5.00	2.92	1.16
Appreciation of others' strengths	3	2.00	5.00	2.93	1.18
Contributions and teach ability	3	1.00	5.00	2.60	0.98
Decent work	16	1.60	4.80	2.94	0.86
Work rewards	4	1.00	5.00	2.15	0.97
Work position	3	1.00	5.00	2.34	0.98
Work atmosphere	3	1.00	5.00	2.48	1.11
Work development	3	1.00	5.00	2.40	1.07
Work recognition	3	1.00	5.00	2.48	1.22
Grit	8	1.00	5.00	2.78	0.89

Abbreviation: SD = standard deviation.

**Table 3:** Correlation between study variables among participants (*N* = 454).

Variables	1	2	3
Humble leadership	1		
Decent work	0.38^∗∗^ (*p* < 0.001)	1	
Grit	0.36^∗∗^ (*p* < 0.001)	0.42^∗∗^ (*p* < 0.001)	1

*Note: r* = Pearson correlation.

^∗∗^Correlation is highly significant at the 0.01 level (2-tailed).

**Table 4:** Mediating effect of decent work between humble leadership and nurses' grit (*n* = 454).

	(*B*)	CI 95%	*t*	*p*
Direct effect				
Humble leadership ⟶ decent work	0.488	(0.38–0.59)	8.89	< 0.001^∗∗^
Humble leadership ⟶ nurses' grit	0.308	(0.19–0.42)	5.26	< 0.001^∗∗^
Decent work ⟶ nurses' grit	0.342	(0.25–.43)	7.35	< 0.001^∗∗^
Indirect effect				
Humble leadership ⟶ decent work ⟶ nurses' grit	0.267	(0.10–0.23)	4.96	< 0.001^∗∗^
Total effect				
Humble leadership ⟶ nurses' grit	0.475	(0.36–0.58)	8.55	< 0.001^∗∗^

^∗∗^Correlation is highly significant at the 0.01 level (2-tailed).

## Data Availability

The data that support the findings of this study are available on request from the corresponding author. The data are not publicly available due to privacy or ethical restrictions.
